# Intensive Care Unit–Specific Virtual Reality for Critically Ill Patients With COVID-19: Multicenter Randomized Controlled Trial

**DOI:** 10.2196/32368

**Published:** 2022-01-31

**Authors:** Johan H Vlake, Jasper van Bommel, Evert-Jan Wils, Joe Bienvenu, Merel E Hellemons, Tim IM Korevaar, Anna FC Schut, Joost AM Labout, Lois LH Schreuder, Marten P van Bavel, Diederik Gommers, Michel E van Genderen

**Affiliations:** 1 Department of Intensive Care, Erasmus MC Rotterdam Netherlands; 2 Department of Intensive Care, Franciscus Gasthuis & Vlietland Rotterdam Netherlands; 3 Department of Psychiatry and Behavioral Sciences John Hopkins University School of Medicine Baltimore, MD United States; 4 Department of Pulmonology, Erasmus MC Rotterdam Netherlands; 5 Department of Internal Medicine, Academic Centre for Thyroid Diseases Erasmus MC Rotterdam Netherlands; 6 Department of Intensive Care, Ikazia hospital Rotterdam Netherlands; 7 Department of Intensive Care, Maasstad hospital Rotterdam Netherlands

**Keywords:** SARS-CoV-2, intensive care, post-intensive care syndrome, virtual reality, quality of life, satisfaction, COVID-19

## Abstract

**Background:**

Although psychological sequelae after intensive care unit (ICU) treatment are considered quite intrusive, robustly effective interventions to treat or prevent these long-term sequelae are lacking. Recently, it was demonstrated that ICU-specific virtual reality (ICU-VR) is a feasible and acceptable intervention with potential mental health benefits. However, its effect on mental health and ICU aftercare in COVID-19 ICU survivors is unknown.

**Objective:**

This study aimed to explore the effects of ICU-VR on mental health and on patients’ perceived quality of, satisfaction with, and rating of ICU aftercare among COVID-19 ICU survivors.

**Methods:**

This was a multicenter randomized controlled trial. Patients were randomized to either the ICU-VR (intervention) or the control group. All patients were invited to an COVID-19 post-ICU follow-up clinic 3 months after hospital discharge, during which patients in the intervention group received ICU-VR. One month and 3 months later (4 and 6 months after hospital discharge), mental health, quality of life, perceived quality, satisfaction with, and rating of ICU aftercare were scored using questionnaires.

**Results:**

Eighty-nine patients (median age 58 years; 63 males, 70%) were included. The prevalence and severity of psychological distress were limited throughout follow-up, and no differences in psychological distress or quality of life were observed between the groups. ICU-VR improved satisfaction with (mean score 8.7, SD 1.6 vs 7.6, SD 1.6 [ICU-VR vs control]; *t*_64_=–2.82, *P*=.006) and overall rating of ICU aftercare (mean overall rating of aftercare 8.9, SD 0.9 vs 7.8, SD 1.7 [ICU-VR vs control]; *t*_64_=–3.25; *P*=.002) compared to controls. ICU-VR added to the quality of ICU aftercare according to 81% of the patients, and all patients would recommend ICU-VR to other ICU survivors.

**Conclusions:**

ICU-VR is a feasible and acceptable innovative method to improve satisfaction with and rating of ICU aftercare and adds to its perceived quality. We observed a low prevalence of psychological distress after ICU treatment for COVID-19, and ICU-VR did not improve psychological recovery or quality of life. Future research is needed to confirm our results in other critical illness survivors to potentially facilitate ICU-VR’s widespread availability and application during follow-up.

**Trial Registration:**

Netherlands Trial Register NL8835; https://www.trialregister.nl/trial/8835

**International Registered Report Identifier (IRRID):**

RR2-10.1186/s13063-021-05271-z

## Introduction

The increase in the survival of critically ill patients admitted to the intensive care unit (ICU) in the last few decades has revealed the effect of ICU treatment on quality of life [1-3]. Up to one-third of “general” ICU survivors experience a poor quality of life, predominantly owing to psychological sequelae such as anxiety, depression, and posttraumatic stress disorder (PTSD) [4-7]. These psychological impairments comprise the psychological component of the postintensive care syndrome (PICS); they are common and can last months to even years after patient ICU discharge [4,8,9]. Consequently, there is a need for post-ICU care.

As the demand for ICU beds and critical care services has skyrocketed during the current COVID-19 outbreak, so would that of ICU aftercare. As such, health care services will have to adapt rapidly to an anticipated surge of post-ICU care, and this will place an enormous strain on acute services [10]. The current pandemic is highlighting the urgency for a multimodal follow-up program and the need for patient-focused innovative solutions [11]. Difficulty accessing in-person clinics is a key barrier in the development of an ICU follow-up program and is probably hindered more owing to current COVID-19 regulations [12]. While post-ICU care has been recognized as a fundamental part of ICU care by the critical care community, effective interventions and guidelines are lacking, and evidence for the effectiveness of post-ICU programs has not been established [4,13,14]. The unmet need for information and the increasing importance of satisfaction as an important quality indicator are two major denominators that could explain the aforementioned and should, as such, be taken into account in developing post-ICU care in the current era [15,16].

Recovery from COVID-19 could have the same multifaceted problems that occur after sepsis and other critical illnesses [17]. Recently, virtual reality (VR) was demonstrated to be a useful technique to improve post-ICU mental health in sepsis survivors and could also be safely used and implemented in post-ICU COVID-19 care [18-20]. As such, we hypothesized that an ICU-specific virtual reality (ICU-VR) intervention could improve the satisfaction with and rating of ICU aftercare and could contribute to psychological recovery. The aims of this study were therefore to explore the effects of ICU-VR on mental health and on patients’ perceived quality and satisfaction with and rating of ICU aftercare among COVID-19 ICU survivors.

## Methods

### Study Design

This multicenter, open-label, randomized controlled trial was conducted in a university teaching hospital and in 3 university-affiliated secondary care hospitals. Patients were included from June 2020 to February 2021 and were followed-up for 6 consecutive months. The study protocol was approved by the Medical Ethics Committee of the Erasmus Medical Centre, Rotterdam, and the participating centers’ institutional review boards (NL73667.078.20, approved June 10, 2020) and has previously been published [21].

### Participants

All consecutive adult (≥18 years) patients who were treated in an ICU of one of the participating hospitals and visited the COVID-19 post-ICU follow-up clinic were eligible for inclusion. COVID-19 was diagnosed on the basis of a positive finding on reverse transcription–polymerase chain reaction (RT–PCR) for SARS-CoV-2. Exclusion criteria were primary neurological impairments or documented active psychiatric diseases, an inability to understand the Dutch language, absence of a formal home address, and participation in other interventional trials that could confound the primary outcome. Patients and the public were not involved in the design, conduct, reporting, or dissemination plans of this study. A former ICU patient was involved in the development of the ICU-VR intervention.

### Randomization and Masking

Patients were randomly assigned to either the ICU-VR (intervention) group or the control group at a 1:1 ratio, using a centralized internet-based randomization procedure by the study site’s principal investigator or a representative (Castor Electronic Data Capture [EDC]). Patients were randomized in a simple manner without stratification. The investigators were unaware of the assignment sequence. Owing to the nature of the intervention, blinding of patients and investigators was not possible.

### Intervention

All patients were invited to a COVID-19 post-ICU follow-up clinic as part of regional standard care. During this visit, patients had a 60-minute-long consultation with an intensivist and an ICU nurse, during which the ICU treatment was reviewed, and patients were screened for PICS-related impairments and referred to an appropriate health care worker, if appropriate.

Patients in the ICU-VR group received the ICU-VR intervention once during this visit. ICU-VR is explained in depth elsewhere. In short, it was developed by an interdisciplinary team that included intensivists, ICU nurses, a psychologist, a psychiatrist, an investigator, and former ICU patients and was previously demonstrated to be safe and feasible [18,22]. ICU-VR consists of a 14-minute-long informational video that can be watched using VR, in which the patient is exposed to the ICU environment and receives voice-over explanations regarding different facets of the surrounding ICU and ICU treatment. ICU-VR consists of 6 scenes: (1) The ICU physician and nurse welcome the patient in front of the ICU. After being brought to and installed in the ICU, explanations are given (2) about the surveillance monitor, medication pumps, intubation (including tracheal tube suction), mechanical ventilation, and prone positioning; (3) about intravenous drips and lines and tracheotomy, including its procedures; (4) about the treatment team taking care of the patient; (5) about isolation measures and personal protection equipment; and (6) about COVID-19 [19,21]. The script and the YouTube version can be found elsewhere [21,23]. The ICU-VR intervention was watched using head-mounted display–VR glasses (Oculus Go) in combination with headphones.

### Study Procedures

All COVID-19 ICU survivors were invited to the hospital’s COVID-19 post-ICU follow-up clinic 3 months after hospital discharge as part of regional standard care. One month prior to this visit, eligible patients were sent a study information brochure, and 2 weeks later, patients were contacted by telephone by a member of the study team to explain the study procedures. During their follow-up clinic visit, consent was obtained, and patients were randomized.

Patients randomized to the ICU-VR group received the ICU-VR intervention once during the concordant follow-up clinic visit, whereas patients randomized to the control group did not receive ICU-VR. Aside from the ICU-VR intervention, there were no differences between the study groups. These results are part of a larger study evaluating the long-term effects of ICU-VR after 6 months and the effect of VR crossover [21].

Prior to the follow-up clinic visit and 4 and 6 months after hospital discharge—that is, 1 and 3 months after the COVID-19 post-ICU follow-up clinic visit—psychological distress and quality of life were assessed. Six months after hospital discharge, all patients were asked about their satisfaction with and rating of ICU care and aftercare, and patients in the intervention group were asked about their perspectives on ICU-VR.

### Outcomes

Primary outcomes were PICS-related psychological distress and quality of life up to 6 months after hospital discharge and were mandatory parts of the questionnaire.

Psychological distress was expressed as the prevalence and severity of PTSD, anxiety, and depression-related symptoms assessed using the Impact of Event Scale-Revised (IES-R; PTSD) and the Hospital Anxiety and Depression Scale (HADS; anxiety and depression), respectively [24,25]. The IES-R is a self-reported measurement consisting of 22 items that assesses subjective distress caused by a traumatic event and has previously been validated in ICU survivors [24,26,27]. It provides a total score ranging from 0 to 88, with higher scores indicating more severe symptoms. It also provides subscale scores to assess symptoms of intrusion, avoidance, and hyperarousal, which is the sum of all items in each section. An IES-R total score of ≥34 is considered the optimal cutoff for PTSD [28]. The HADS consists of 14 items and is commonly used to determine the levels of anxiety and depression that a patient is experiencing and has been validated in critical illness survivors [29-31]. Seven of the items relate to anxiety, 7 relate to depression, and each question is answered on a 4-point Likert scale. A sum score of ≥8 (ranging from 0 to 21, with higher scores indicating more severe symptoms) on either the depression or the anxiety subscale, is classified as clinically meaningful depression and anxiety, respectively [29].

Quality of life was assessed using the Short-Form 36 (SF-36) and the European Quality of Life, 5 Dimensions (EQ-5D) questionnaires [32,33]. The EQ-5D and SF-36 have been validated and tested in the ICU and have been recommended for use in critical care medicine [34-38]. The EQ-5D measures quality of life in 5 dimensions (mobility, self-care, usual activities, pain or discomfort, and anxiety or depression). In each domain, patients are asked if they experience no, slight, moderate, severe, or extreme problems, from which the weight of a health state can be computed, ranging from –0.446 (worst quality of life) to 1.000 (best quality of life) [39]. Additionally, patients score their current subjective health on a visual analog scale, ranging from 0 (worst health imaginable) to 100 (best health imaginable). SF-36 is a 36-item, patient-reported survey of health and health-related quality of life (HRQoL). It consists of 8 scaled scores, which are the weighted sums of the questions in their section, and a scale for health change. Each scale is directly transformed to a scale ranging from 0 (worst score) to 100 (best score) on the assumption that each question carries an equal weight. The 8 sections are physical functioning, social functioning, physical role functioning, emotional role functioning, emotional well-being, vitality, bodily pain, and general health perception [40]. In addition to these scales, mental and physical component scores can be calculated, which represent a patient’s mental and physical health state. These scores are computed so that the mean is 50 (SD 10) for the general population [41,42].

Patients’ perceived quality of and patients’ satisfaction with and rating of ICU aftercare were assessed using a novel questionnaire, and the questions were nonmandatory to answer. The questionnaire was based on the Patient Satisfaction Questionnaire and Family Satisfaction with ICU Care tools, altered to the needs of this study [43-45]. This questionnaire consisted of 21 items and was categorized into five sections: perspectives on the added value of ICU-VR to ICU care and ICU aftercare (8 questions), perspectives on the timing and number of sessions (3 questions), overall perspective on the ICU-VR intervention (3 questions), perspectives on the content of the ICU-VR intervention (3 questions), and perspectives on the effect of the ICU-VR intervention (4 questions; Multimedia Appendix 1). The first section was (partly) answered by all patients, irrespective of the randomization allocation, and the other sections were answered only by patients randomized to the ICU-VR group. All questions could be answered on a 10-point Likert scale, ranging from 1 (not at all) to 10 (very much), except for perspectives on the timing and number of sessions. The questionnaire was administered by telephone.

Baseline characteristics and survival were determined through patient record analysis. Additional demographics, such as educational level and preadmission employment status, were assessed using follow-up questionnaires.

### Statistical Analysis

Based on a previous pilot study examining the feasibility, safety, and clinical relevance of sepsis ICU-VR (Cohen *d* effect size=0.77), we assumed the effect estimates of ICU-VR to be similar in this study [20]. Using a 2-sided α value of .05, a power of .80, a 1:1 randomization, and an expected loss to follow-up of 20%, we aimed to include a minimum of 80 patients, with 40 patients in each study group.

Baseline demographics and treatment-related characteristics were quantified using descriptive statistics. Continuous variables are expressed as median (IQR) or mean (SD) values, depending on their distribution. Categorical variables are presented as absolute numbers and relative frequencies.

Differences between study groups in continuous variables, such as the IES-R sum score, the HADS anxiety and depression scores, the SF-36 subscales and the EQ-5D utility score, at several follow-up time points were analyzed using a mixed-effects linear regression model with a random intercept for each study site. Differences in continuous outcomes at the 3-month follow-up time point were adjusted by adding the 3-month outcome as an independent variable in the mixed-effects linear regression model. Patients were categorized on the basis of clinically meaningful cutoffs for the IES-R sum score and the HADS anxiety and depression scores. Differences in categorical variables between study groups at several follow-up time points were analyzed using a mixed-effects logistic regression model with a random effect for each site. Differences in categorical outcomes at the 3-month follow-up time point were adjusted by adding the 3-month outcome as an independent variable in the mixed-effects logistic regression model. Differences in continuous or categorical variables throughout follow-up were analyzed using a mixed-effects linear or logistic regression model with time, randomization, and a random intercept or slope for each individual and each study site as appropriate. Differences in linear or categorical outcomes at the 3-month follow-up time point were added to the mixed-effects linear or logistic model as independent variables to adjust for that difference.

Outcomes of the mixed-effects linear regression models are reported as coefficient (95% CI) values, which implies the estimated mean difference, and outcomes of the mixed-effects logistic regression models are reported as odds ratios (ORs) with corresponding 95% CI values.

All data were gathered using Castor EDC. All analyses were performed using SPSS (version 24.0; SPSS Inc) and R for Statistics (R Foundation for Statistical Computing). A *P* value of ≤.05 was considered statistically significant.

### Data Sharing

All data sets created during this study are available upon reasonable request by the corresponding author.

## Results

### Results Overview

**Figure 1:**
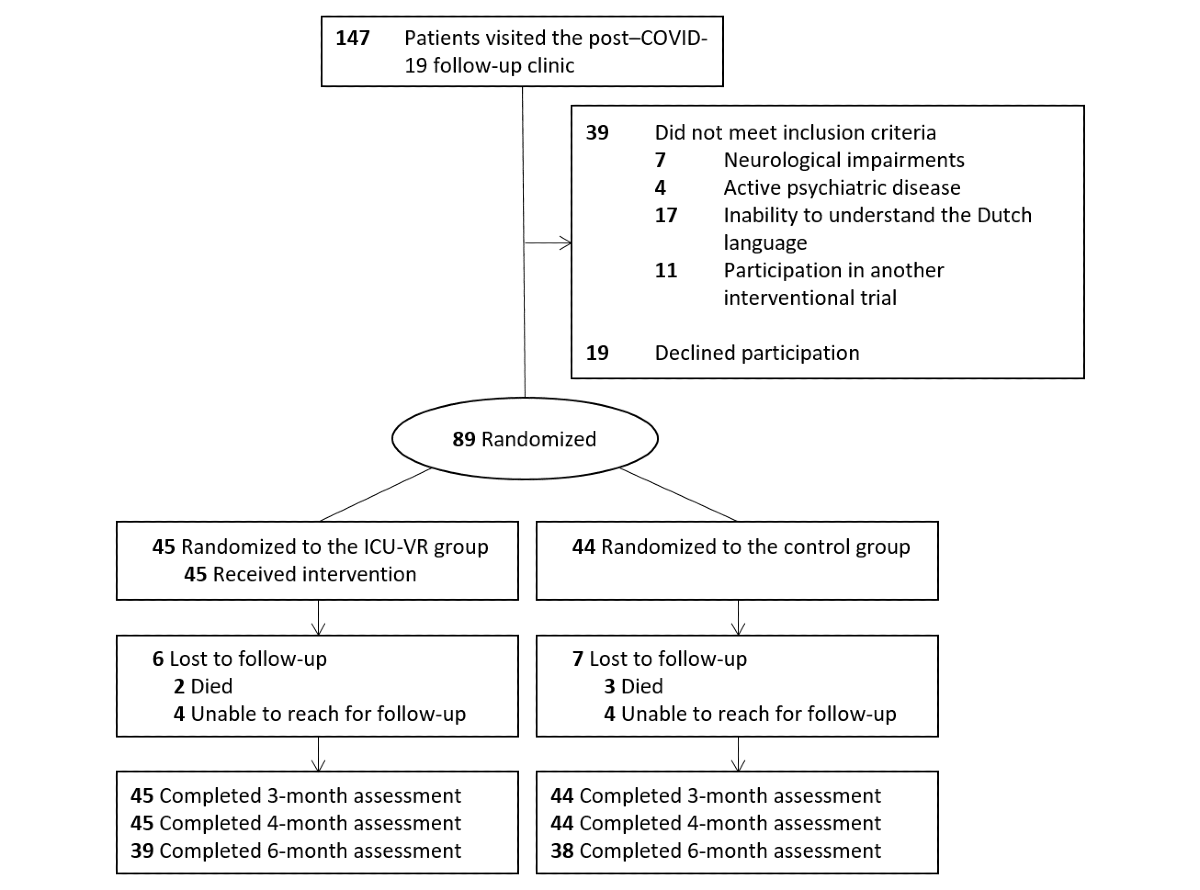
CONSORT (Consolidated Standards of Reporting Trials) flow diagram of the study. ICU-VR: intensive care unit–specific virtual reality.

A total of 147 patients visited the COVID-19 post-ICU follow-up clinic, of whom 89 were enrolled (inclusion rate: 61%): 45 patients in the ICU-VR group and 44 patients in the control group (Figure 1). All patients in the ICU-VR group completed the ICU-VR intervention, and no adverse events were reported. Baseline demographics and treatment-related characteristics were well balanced between groups (Table 1). The mean age was 58 (SD 11) years, 63 patients (71%) were male, and the median ICU length of stay was 17 (IQR 9-29) days. Table 1 shows baseline demographics and treatment-related characteristics.

**Table 1:** Baseline demographics and treatment-related characteristics.

Characteristics^a^					ICU-VR^b^ group (intervention) (n*=*45)			Control group (n=44)
**Baseline demographics**								
	Age (years), median (IQR)			61 (54-65)			59 (51-65)	
	Males, n (%)			35 (78)			28 (36)	
	**BMI, median (IQR)**			27.6 (25.3-31.1)			28.0 (25.3-31.2)	
		Participants with a BMI of >30, n (%)	14 (31)			18 (41)		
	**Educational level, n (%)**							
		Primary education	14 (31)			13 (30)		
		Intermediate vocational education	15 (33)			20 (46)		
		Higher vocational education	13 (29)			7 (16)		
		Academic education	3 (7)			4 (9)		
	Employment status, employed, n (%)			23 (51)			21 (48)	
**Treatment-related characteristics**								
	Length of stay in the intensive care unit (days), median (IQR)			14 (9-25)			14 (7-28)	
	Length of hospital stay (days), median (IQR)			22 (12-32)			24 (13-40)	
	**Mechanical ventilation, n (%)**			41 (91)			38 (86)	
		Duration (hours), median (IQR)	227 (169-343)			383 (206-465)		
		Highest positive end-respiratory pressure (cm H_2_O), median (IQR)	21 (17-28)			20 (16-25)		
		Lowest fraction of inspired oxygen (%), median (IQR)	28 (24-30)			25 (22-30)		
		Lowest ratio of arterial oxygen (mm Hg), median (IQR)	0.11 (0.09-0.23)			0.11 (0.09-0.18)		
		Prone positioning, n (%)	35 (77)			36 (82)		
	**Medication**							
		Received noradrenaline, n (%)	37 (82)			35 (80)		
		Noradrenaline dose (µg/kg/minute), median (IQR)	0.17 (0.10-0.30)			0.14 (0.08-0.29)		
		Duration of noradrenaline use (hours), median (IQR)	186 (32-249)			167 (96-349)		
		Received midazolam, n (%)	35 (78)			33 (75)		
		Midazolam dose (mg/kg/hour), median (IQR)	0.59 (0.43-0.71)			0.51 (0.39-0.66)		
		Duration of midazolam use (hours), median (IQR)	20 (13-93)			20 (13-36)		
		Received remifentanil, n (%)	32 (71)			35 (80)		
		Remifentanil dose (µg/kg/hour), median (IQR)	14 (10-16)			14 (6-18)		
		Duration of remifentanil use (hours), median (IQR)	33 (22-80)			32 (23-72)		
		Received sufentanil, n (%)	26 (58)			28 (63)		
		Sufentanil dose (µg/kg/hour), median (IQR)	0.55 (0.34-0.83)			0.60 (0.38-0.70)		
		Duration of sufentanil use (hours), median (IQR)	8 (1-13)			10 (6-14)		
		Received rocuronium, n (%)	22 (49)			16 (36)		
		Rocuronium dose (mg/kg/hour), median (IQR)	0.39 (0.05-0.77)			0.32 (0.01-0.60)		
		Duration of rocuronium use (hours), median (IQR)	22 (0-28)			17 (0-22)		
	**Illness severity scores**							
		Simplified Acute Physiology Score (version 2), median (IQR)	31 (26-36)			31 (26-35)		
		Acute Physiology and Chronic Health Evaluation (version 4) score, median (IQR)	49 (38-60)			49 (42-59)		
		Admission Sequential Organ Failure Assessment score, median (IQR)	2 (1-6)			2 (1-4)		
		Highest Sequential Organ Failure Assessment score, median (IQR)	8 (6-10)			7 (6-9)		

^a^Baseline demographics and treatment-related characteristics were obtained at 3 months after hospital discharge via digital patient records.

^b^ICU-VR: intensive care unit-specific virtual reality.

### Psychological Component of the PICS

At the 3-month follow-up time point, a total of 31 of 89 patients (34%) reported psychologic distress, with 10 patients (22%) in the ICU-VR group and 21 patients (47%) in the control group (OR 3.5, 95% CI 1.4-8.9, *P*<.01). At 4 months, 38 patients (43%) reported psychological distress, with 12 patients (27%) in the ICU-VR group and 26 patients (59%) in the control group (OR 3.0, 95% CI 0.8-11.9, *P*=.11). At 6 months, 24 patients (31%) reported psychological distress, with 9 patients (23%) in the ICU-VR group and 15 patients (39%) in the control group (OR 0.7, 95% CI 0.2-2.9, *P*=.60).

**Figure 2:**
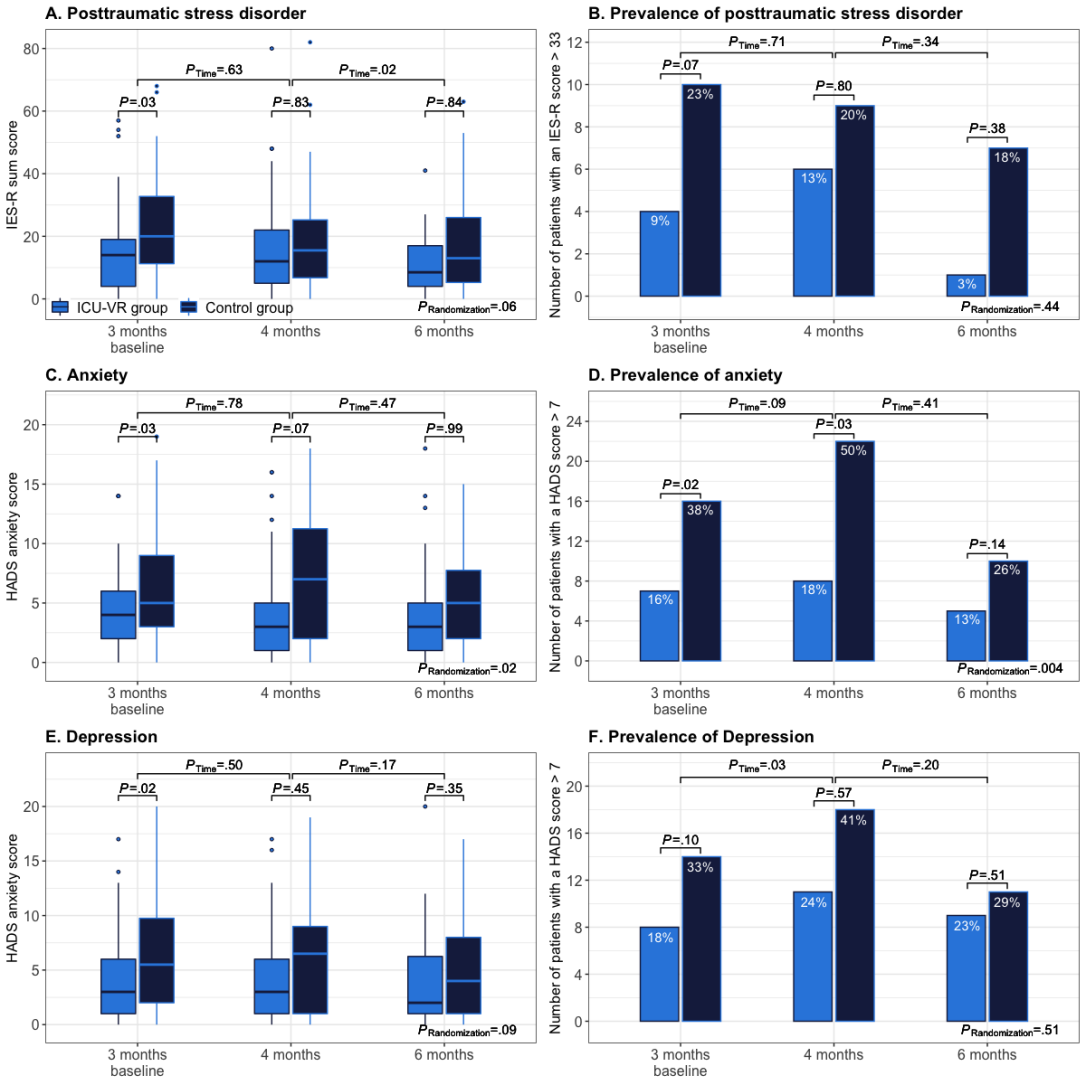
Psychological outcomes. Boxplots of the severity of posttraumatic stress disorder (A), anxiety (C), and depression (E) and bar plots of the prevalence of posttraumatic stress disorder (B), anxiety (D), and depression (F). Posttraumatic stress disorder was assessed using the IES-R, and a sum score of ≥33 was considered as posttraumatic stress disorder being prevalent; anxiety and depression were assessed using the HADS, and a score of ≥8 on either the anxiety or depression scale was considered anxiety and depression being prevalent, respectively. Differences between randomization groups at each follow-up time point and between follow-up time points (p, Time) and throughout the follow-up (p, Randomization) were analyzed using mixed-effects linear (severity) or logistic (prevalence) regression models. HADS: Hospital Anxiety and Depression Scale, IES-R: Impact of Event Scale-Revised.

At the 3-month follow-up time point, 4 patients (9%) in the ICU-VR group and 10 patients (22%) in the control group reported probable PTSD (OR 3.2, 95% CI 0.9-11.1, *P*=.07; Figure 2B). During follow-up, no differences were observed in PTSD scores or the proportion of patients who reported probable PTSD between randomization allocations (Figures 2A and 2B). Throughout follow-up, the PTSD score remained similar at 4 months (β=–.60, 95% CI –3.2 to 1.9, *P*=.63) after hospital discharge but improved at 6 months after hospital discharge (β=–3.1, 95% CI –5.8 to –0.4, *P*=.02). However, this improvement was independent of the randomization group (β=5.4, 95% CI –0.2 to 11.1, *P*=.06).

At the 3-month follow-up time point, 7 patients (16%) in the ICU-VR group and 16 patients (38%) in the control group reported probable anxiety (OR 3.3, 95% CI 1.2-9.3, *P*=.02; Figure 2D). Four months after hospital discharge, ICU-VR resulted in fewer patients with probable anxiety (n=8, 18% vs 22, 50%; OR 3.8, 95% CI 1.1-12.7; *P*=.03; Figure 2C) but not lower anxiety scores (median HADS anxiety score 3, IQR 1-5 vs 7, IQR 2-11; β=1.4, 95% CI –0.1 to 3.0; *P*=.07). There were no differences at 4 or 6 months after hospital discharge (Figures 2C and 2D). No natural decline in anxiety was observed, and the severity of anxiety and the prevalence of probable anxiety were lower in the ICU-VR group throughout the follow-up.

At the 3-month follow-up time point, 8 (18%) patients in the ICU-VR group and 14 (33%) patients in the control group reported probable depression (OR 2.3, 95% CI 0.9-6.3, *P*=.10; Figure 2F). Throughout the follow-up, no difference in the depression scores or the proportion of patients reporting probable depression was observed (Figures 2E and 2F). The severity of depression remained similar throughout the follow-up period.

### Health-Related Quality of Life

**Figure 3:**
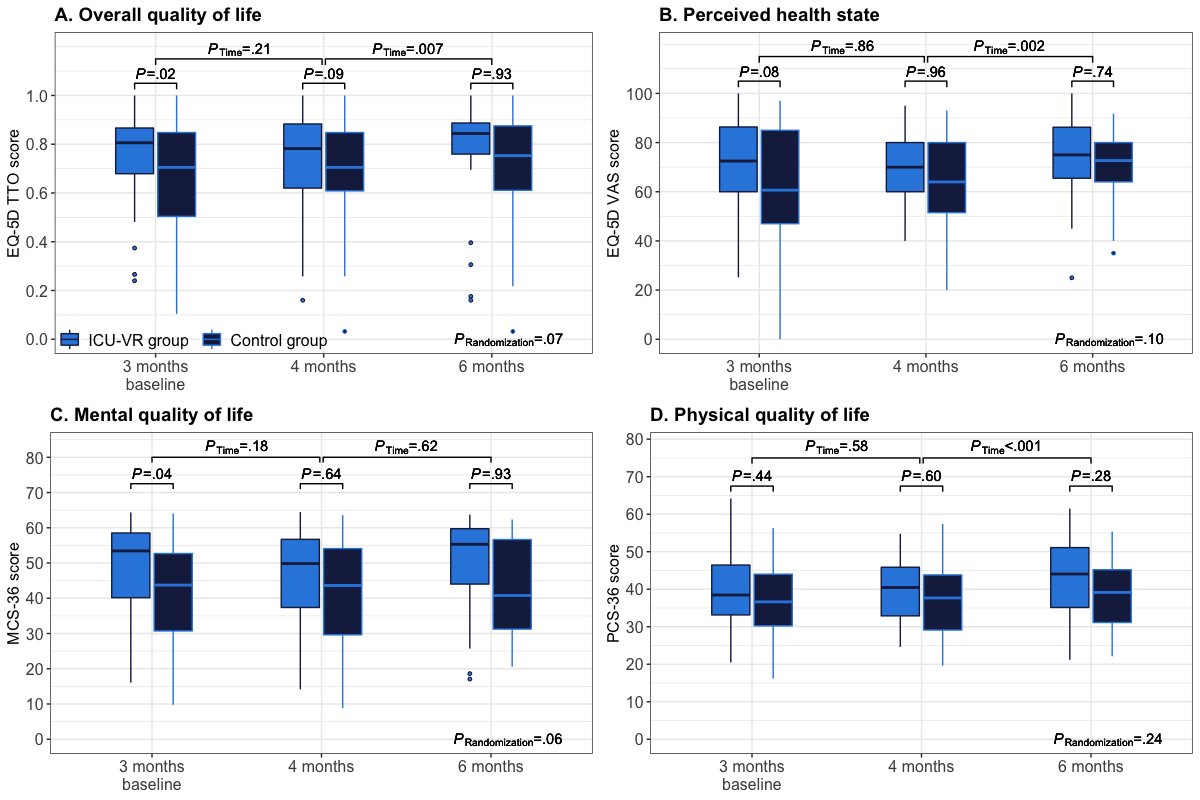
Quality of life outcomes. Boxplots of the overall quality of life (A), perceived health state (B), mental quality of life (C), and physical quality of life (D). Overall quality of life was expressed as the EQ-5D TTO score, the perceived health state as the EQ-5D VAS score, and the mental and physical quality of life as the mental and physical component scales of the SF-36, respectively. Differences between randomization groups at each follow-up time point and between follow-up time points (p, Time) and throughout the follow-up (p, Randomization) were analyzed using mixed-effects linear (severity) or logistic (prevalence) regression models. EQ-5D: European Quality of Life, 5 dimensions, ICU-VR: intensive care unit–virtual reality, MCS-36: Mental Component Summary, 36 items, PCS-36: Physical Component Summary, 36 items, TTO: trade time-off, VAS: visual analog scale.

The overall health-related quality of life, mental health–related quality of life, and physical health–related quality of life are depicted in Figure 3**.** Throughout the follow-up period, the overall, mental, and physical HRQoL remained similar until 4 months but improved at 6 months after hospital discharge, while overall quality of life, outcomes of individual EQ-5D domains, and subscales of the SF-36 score differed between groups during the follow-up period (Figures 3A-D, Multimedia Appendix 2 and Multimedia Appendix 3).

### Perspectives on ICU-VR

**Figure 4:**
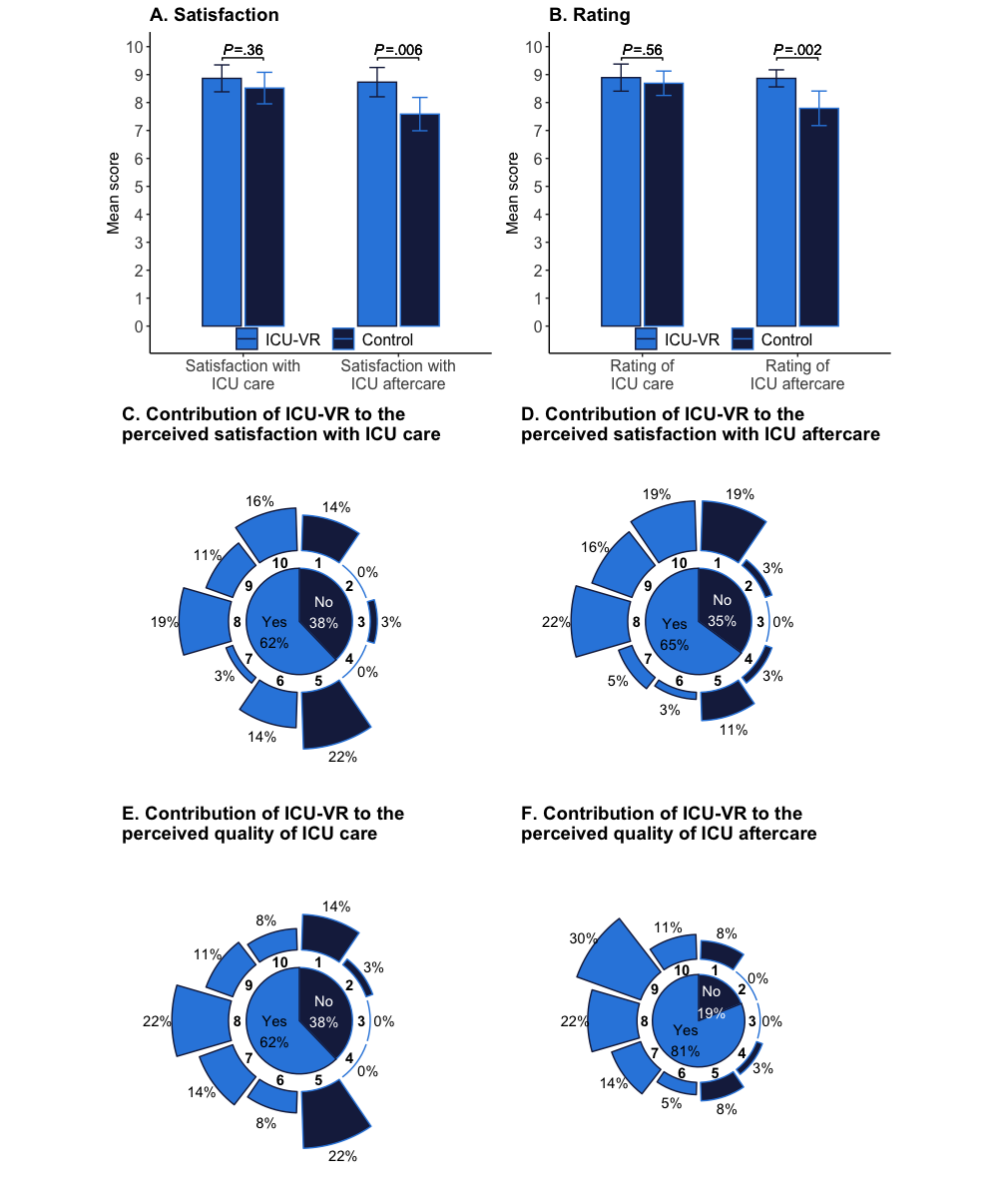
Perspectives on ICU-VR. Bar charts of the mean satisfaction score (A) and rating (B) of ICU care (left) and ICU aftercare (right) in the ICU-VR and control group, wherein the error bars indicate the 95% CI of the scores. The contribution of ICU-VR to the perceived satisfaction with ICU care (C) and ICU aftercare (D) and the contribution of ICU-VR to the perceived quality of ICU care (E) and ICU aftercare (F) are presented as combined pie/bar charts, indicating the percentage of patients in the ICU-VR group who gave a score above 5 (inner circle) and the percentage of patients in the ICU-VR group giving a certain score (outer circle). ICU: intensive care unit, ICU-VR: intensive care unit–virtual reality.

In total, 37 patients (84%) in the ICU-VR group and 32 patients (71%) in the control group gave their perspective about the intervention and the received care and aftercare (Figure 4 and Multimedia Appendix 4)**.** Patients in the intervention group were more satisfied with the ICU aftercare (mean score: 8.7, SD 1.6 vs 7.6, SD 1.6 [ICU-VR vs control], *t*_64_=–2.82, *P*=.006) but not with the ICU care (mean score 8.9, SD 1.5 vs 8.5, SD 1.5 [ICU-VR vs control], *t*_64_=–0.92; *P*=.36; Figure 4A). Additionally, patients in the intervention group rated the ICU aftercare higher (mean overall rating of aftercare 8.9, SD 0.9 vs 7.8, SD 1.7 [ICU-VR vs control], *t*_64_=–3.25; *P*=.002) but not the ICU care (mean score 8.9, SD 1.5 vs 8.7, SD 1.2 [ICU-VR vs control], *t*_64_=–0.59; *P*=.56; Figure 4B). ICU-VR added to the satisfaction of ICU care according to 62% of patients (Figure 4C), satisfaction with ICU aftercare according to 65% of patients (Figure 4D), quality of ICU care according to 62% patients (Figure 4E), and quality of ICU aftercare according to 81% of patients in the ICU-VR group (Figure 4F).

Patients in the intervention group assigned a mean score of 8.7 (SD 1.0) out of 10 to the ICU-VR, on a Likert scale, and stated that ICU-VR improved their understanding of ICU treatment (score>5, 76%; mean score 7.2, SD 2.5) and decreased their frightening memories (score>5, n=24-37, 65%; mean score 6.6, SD 2.8; Multimedia Appendix 4).

## Discussion

### Principal Findings

We observed that ICU-VR improved patients’ perceived quality of, satisfaction with, and rating of ICU aftercare among COVID-19 ICU survivors. This method is feasible, acceptable, and innovative and could be implemented in regional ICU aftercare. Our results also demonstrate that approximately 31% of COVID-19 ICU survivors experienced decreased mental health in terms of psychological distress up to 6 months after hospital discharge and that ICU-VR did not improve psychological recovery or quality of life**.**

In contrast to our previous findings regarding patients with sepsis and a recent COVID-19 case report, we did not observe improved mental health or quality of life in the ICU-VR group [19,20]. In contrast to this study, we provided ICU-VR earlier post ICU admission (median 7-8 days) in our previous study. Notably, the patient with COVID-19 in our case report and those in the sepsis study had robust responses in terms of psychological distress symptoms, including PTSD. The COVID-19 critical illness survivors in this study received ICU-VR much later (3 months after hospital discharge). Therefore, the timing of the ICU-VR intervention could be important for its therapeutic effect. Although 3 months after hospital discharge is a clinically feasible time point, it can be argued that PTSD and anxiety, at that moment, have already fully developed, and treatment of fully established psychiatric disorders may require more complex treatment strategies. When ICU-VR is offered soon after ICU discharge; that is, in the initial few weeks patients are still processing what happened to them, and ICU-VR could be a valuable adjunct to improve factual recall and decrease frightening memories. In future studies, the timing of ICU-VR and the number of sessions needed should be further investigated.

The number of desired or needed VR sessions remains a matter of debate, and no study has determined the optimal number of sessions after ICU admission. Although an average of 8-14 sessions is used in nonhospital settings, we previously demonstrated that sepsis survivors desire a median of only one session [20,46,47]. In this study, more than half of the patients did not desire the ICU-VR intervention multiple times, although there was substantial interpatient variability. An important difference between the current study and the sepsis trial is that in the sepsis trial, patients could self-determine how many sessions they desired, and this could have potentially increased the effectiveness. Therefore, a more patient-centered approach instead of a prespecified number of times might be more suitable, though guidelines are currently lacking.

Additionally, we observed lower overall incidence rates of PTSD (22% vs 11%), anxiety (46% vs 21%), and depression (41% vs 18%) at 3 months compared to a recent nationwide study in the United Kingdom, which included all patients who received at least 24 hours of ICU treatment, and compared to previously observed studies involving patients with COVID-19, acute lung injury (and acute respiratory distress syndrome) survivors, and a Dutch cohort of critical illness survivors [8,9,16,48-50]. Importantly, our power calculation was based on the prevalence rates of psychological distress. This lower incidence might explain the lack of ICU-VR effectiveness for this item. The lack of predisposing factors (such as a pre-existing cognitive impairment) could possibly explain the low prevalence of PTSD and depression in the current population [51-53].

Satisfaction during and after ICU admission is increasingly becoming an issue of interest considering that low satisfaction negatively impacts psychological sequelae after critical care [54]. Evidence suggests that patients generally indicate that they are satisfied with ICU care [55,56]. We found similarly high levels of reported satisfaction and showed that despite these high numbers, ICU-VR improved satisfaction, ratings, and perceived quality of ICU aftercare. Moreover, 100% would recommend ICU-VR to other patients. This seems to suggest that satisfaction with patient care does not imply that there are no problems regarding some aspects of their inpatient experience or that they fully comprehended all ICU-related information. Our findings actually confirm the results from a recent review, which concluded that patients’ support after ICU admission is multifaceted and varies across several transition points after ICU discharge [57]. An analogy can be made to civil aviation, where satisfaction may be high, but customers still complain about specific aspects of the service [58]. ICU-VR could therefore serve as an additional modality to fulfill several individual patient needs during the transition from ICU to home. Additionally, despite the lack of a successful effect on “traditional” measurements, such as the psychological questionnaires in this study, more than half of the patients experienced a decrease in frightening memories. Therefore, ICU aftercare might be more complex than we thought and may require a more patient-centered approach for measuring the results of novel intervention methods.

### Limitations

Several study limitations should be acknowledged. First, despite our randomization procedure, there were statistically significant differences in primary outcome measures between groups at the 3-month follow-up time point. To ensure that no effect was overestimated, we adjusted our outcomes for the 3-month follow-up time point outcomes by adding them as independent predictors to our regression models. Although this difference was unexpected considering the randomization procedure, we could have prevented these differences by stratifying the randomization procedure on the presence of psychological distress at the follow-up time point prior to randomization. In future studies, we should consider this when possible. Second, as both the ICU-VR intervention and the questionnaire were in Dutch, we could only include patients able to understand the Dutch language. This may have resulted in selection bias, and we do not know how ICU-VR performs in nonnative Dutch speakers or if a translated version has an effect in these patients. This is especially of interest as, owing to language restrictions, these patients are expected to understand less of their ICU treatment than native Dutch patients and may therefore benefit more from such an intervention. Third, we used a novel set of questionnaires to assess patient experiences. Although these were based on and altered from the Patient Satisfaction Questionnaire and Family Satisfaction with ICU Care tools, these questionnaires have not yet been validated [43-45].

### Conclusions

In conclusion, ICU-VR is a feasible and acceptable innovative method to improve patient satisfaction with and rating of ICU aftercare and adds to its perceived quality. We observed a low prevalence of psychological distress after COVID-19 ICU treatment, and ICU-VR did not improve psychological recovery or quality of life**.** Future studies should explore ICU-VR’s widespread availability and application during ICU follow-up and should determine whether the timing of ICU-VR impacts its effect on psychological PICS-related sequelae.

## Appendix

Multimedia Appendix 1 Translation of the novel questionnaire assessing ICU care and aftercare satisfaction and global rating, and the perspectives on the ICU-specific virtual reality (ICU-VR) intervention.

Multimedia Appendix 2 Table S1. Outcomes of the individual European Quality of Life, 5 dimensions domains.

Multimedia Appendix 3 Table S2. Subscales of the Short-Form 36 throughout follow-up.

Multimedia Appendix 4 Table S3. Perspectives on the ICU-specific virtual reality intervention.

Multimedia Appendix 5 CONSORT (Consolidated Standards of Reporting Trials) checklist.

## Abbreviations

EDC: electronic data capture
EQ-5D: European Quality of Life, 5 dimensions
HADS: Hospital Anxiety and Depression Scale
HRQoL: health-related quality of life
ICU: intensive care unit
ICU-VR: intensive care unit–specific virtual reality
IES-R: Impact of Event Scale-Revised
OR: odds ratio
PICS: postintensive care syndrome
PTSD: posttraumatic stress disorder
RT–PCR: reverse transcription–polymerase chain reaction
SF-36: Short-Form 36
VR: virtual reality

## References

[1] Cuthbertson BH, Roughton S, Jenkinson D, Maclennan G, Vale L (2010). Quality of life in the five years after intensive care: a cohort study. Crit Care.

[2] Lilly CM, Swami S, Liu X, Riker RR, Badawi O (2017). Five-Year Trends of Critical Care Practice and Outcomes. Chest.

[3] Oeyen SG, Vandijck DM, Benoit DD, Annemans L, Decruyenaere JM (2010). Quality of life after intensive care: a systematic review of the literature. Crit Care Med.

[4] Needham DM, Davidson J, Cohen H, Hopkins RO, Weinert C, Wunsch H, Zawistowski C, Bemis-Dougherty A, Berney SC, Bienvenu OJ, Brady SL, Brodsky MB, Denehy L, Elliott D, Flatley C, Harabin AL, Jones C, Louis D, Meltzer W, Muldoon SR, Palmer JB, Perme C, Robinson M, Schmidt DM, Scruth E, Spill GR, Storey CP, Render M, Votto J, Harvey MA (2012). Improving long-term outcomes after discharge from intensive care unit: report from a stakeholders' conference. Crit Care Med.

[5] Kerckhoffs MC, Kosasi FFL, Soliman IW, van Delden JJM, Cremer OL, de Lange DW, Slooter AJC, Kesecioglu J, van Dijk D (2019). Determinants of self-reported unacceptable outcome of intensive care treatment 1 year after discharge. Intensive Care Med.

[6] Jackson JC, Pandharipande PP, Girard TD, Brummel NE, Thompson JL, Hughes CG, Pun BT, Vasilevskis EE, Morandi A, Shintani AK, Hopkins RO, Bernard GR, Dittus RS, Ely EW, Bringing to light the Risk Factors And Incidence of Neuropsychological dysfunction in ICU survivors (BRAIN-ICU) study investigators (2014). Depression, post-traumatic stress disorder, and functional disability in survivors of critical illness in the BRAIN-ICU study: a longitudinal cohort study. Lancet Respir Med.

[7] Marra A, Pandharipande PP, Girard TD, Patel MB, Hughes CG, Jackson JC, Thompson JL, Chandrasekhar R, Ely EW, Brummel NE (2018). Co-Occurrence of Post-Intensive Care Syndrome Problems Among 406 Survivors of Critical Illness. Crit Care Med.

[8] Bienvenu OJ, Friedman LA, Colantuoni E, Dinglas VD, Sepulveda KA, Mendez-Tellez P, Shanholz C, Pronovost PJ, Needham DM (2018). Psychiatric symptoms after acute respiratory distress syndrome: a 5-year longitudinal study. Intensive Care Med.

[9] Parker AM, Sricharoenchai T, Raparla S, Schneck KW, Bienvenu OJ, Needham DM (2015). Posttraumatic stress disorder in critical illness survivors: a metaanalysis. Crit Care Med.

[10] Jaffri A, Jaffri UA (2020). Post-Intensive care syndrome and COVID-19: crisis after a crisis?. Heart Lung.

[11] Simpson R, Robinson L (2020). Rehabilitation After Critical Illness in People With COVID-19 Infection. Am J Phys Med Rehabil.

[12] Haines KJ, McPeake J, Hibbert E, Boehm LM, Aparanji K, Bakhru RN, Bastin AJ, Beesley SJ, Beveridge L, Butcher BW, Drumright K, Eaton TL, Farley T, Firshman P, Fritschle A, Holdsworth C, Hope AA, Johnson A, Kenes MT, Khan BA, Kloos JA, Kross EK, Mactavish P, Meyer J, Montgomery-Yates A, Quasim T, Saft HL, Slack A, Stollings J, Weinhouse G, Whitten J, Netzer G, Hopkins RO, Mikkelsen ME, Iwashyna TJ, Sevin CM (2019). Enablers and Barriers to Implementing ICU Follow-Up Clinics and Peer Support Groups Following Critical Illness: The Thrive Collaboratives. Crit Care Med.

[13] Loudon I (2008). The principle of referral: the gatekeeping role of the GP. Br J Gen Pract.

[14] Schofield-Robinson OJ, Lewis SR, Smith AF, McPeake J, Alderson P (2018). Follow-up services for improving long-term outcomes in intensive care unit (ICU) survivors. Cochrane Database Syst Rev.

[15] Hofhuis JGM, Spronk PE, van Stel HF, Schrijvers AJP, Rommes JH, Bakker J (2008). Experiences of critically ill patients in the ICU. Intensive Crit Care Nurs.

[16] Vlake JH, van Genderen ME, Schut A, Verkade M, Wils E, Gommers D, van Bommel J (2020). Patients suffering from psychological impairments following critical illness are in need of information. J Intensive Care.

[17] Prescott HC, Girard TD (2020). Recovery From Severe COVID-19: Leveraging the Lessons of Survival From Sepsis. JAMA.

[18] Vlake J, Wils E, van Bommel J, Korevaar T, Gommers D, van Genderen ME (2021). Virtual Reality Tailored to the Needs of Post-ICU Patients: A Safety and Immersiveness Study in Healthy Volunteers. Crit Care Explor.

[19] Vlake JH, van Bommel J, Hellemons ME, Wils E, Gommers D, van Genderen ME (2020). Intensive Care Unit-Specific Virtual Reality for Psychological Recovery After ICU Treatment for COVID-19; A Brief Case Report. Front Med (Lausanne).

[20] Vlake J, Van Bommel J, Wils EJ, Korevaar T, Bienvenu OJ, Klijn E, Gommers D, van Genderen ME (2021). Virtual Reality to Improve Sequelae of the Postintensive Care Syndrome: A Multicenter, Randomized Controlled Feasibility Study. Crit Care Explor.

[21] Vlake JH, Van Bommel J, Wils E, Korevaar TIM, Hellemons ME, Schut AFC, Labout JAM, Schreuder LLH, Gommers D, Van Genderen ME (2021). Effect of intensive care unit-specific virtual reality (ICU-VR) to improve psychological well-being and quality of life in COVID-19 ICU survivors: a study protocol for a multicentre, randomized controlled trial. Trials.

[22] Vlake JH, Van Bommel J, Wils E, Korevaar TIM, Bienvenu OJ, Klijn E, Gommers D, van Genderen ME (2021). Virtual Reality to Improve Sequelae of the Postintensive Care Syndrome: A Multicenter, Randomized Controlled Feasibility Study. Crit Care Explor.

[23] Vlake H (2020). Intensive Care Unit-specific Virtual Reality for COVID-19 survivors. YouTube.

[24] Weiss D (2007). The Impact of Event Scale: Revised. Cross-Cultural Assessment of Psychological Trauma and PTSD.

[25] Zigmond AS, Snaith RP (1983). The hospital anxiety and depression scale. Acta Psychiatr Scand.

[26] Bienvenu OJ, Williams JB, Yang A, Hopkins RO, Needham DM (2013). Posttraumatic stress disorder in survivors of acute lung injury: evaluating the Impact of Event Scale-Revised. Chest.

[27] Chan KS, Aronson Friedman L, Bienvenu OJ, Dinglas VD, Cuthbertson BH, Porter R, Jones C, Hopkins RO, Needham DM (2016). Distribution-based estimates of minimal important difference for hospital anxiety and depression scale and impact of event scale-revised in survivors of acute respiratory failure. Gen Hosp Psychiatry.

[28] Creamer M, Bell R, Failla S (2003). Psychometric properties of the Impact of Event Scale - Revised. Behav Res Ther.

[29] Bjelland I, Dahl AA, Haug TT, Neckelmann D (2002). The validity of the Hospital Anxiety and Depression Scale. Journal of Psychosomatic Research.

[30] Sukantarat KT, Williamson RCN, Brett SJ (2007). Psychological assessment of ICU survivors: a comparison between the Hospital Anxiety and Depression scale and the Depression, Anxiety and Stress scale. Anaesthesia.

[31] Jutte JE, Needham DM, Pfoh ER, Bienvenu OJ (2015). Psychometric evaluation of the Hospital Anxiety and Depression Scale 3 months after acute lung injury. J Crit Care.

[32] Ware JE, Sherbourne CD (1992). The MOS 36-item short-form health survey (SF-36). I. Conceptual framework and item selection. Med Care.

[33] EuroQol Group (1990). EuroQol--a new facility for the measurement of health-related quality of life. Health Policy.

[34] Angus DC, Carlet J, 2002 Brussels Roundtable Participants (2003). Surviving intensive care: a report from the 2002 Brussels Roundtable. Intensive Care Med.

[35] Granja C, Teixeira-Pinto A, Costa-Pereira A (2002). Quality of life after intensive care--evaluation with EQ-5D questionnaire. Intensive Care Med.

[36] Badia X, Diaz-Prieto A, Gorriz MT, Herdman M, Torrado H, Farrero E, Cavanilles JM (2001). Using the EuroQol-5D to measure changes in quality of life 12 months after discharge from an intensive care unit. Intensive Care Med.

[37] Heyland DK, Hopman W, Coo H, Tranmer J, McColl MA (2000). Long-term health-related quality of life in survivors of sepsis. Short Form 36: a valid and reliable measure of health-related quality of life. Crit Care Med.

[38] Chrispin PS, Scotton H, Rogers J, Lloyd D, Ridley SA (1997). Short Form 36 in the intensive care unit: assessment of acceptability, reliability and validity of the questionnaire. Anaesthesia.

[39] M Versteegh M, M Vermeulen K, M A A Evers S, de Wit GA, Prenger R, A Stolk E (2016). Dutch Tariff for the Five-Level Version of EQ-5D. Value Health.

[40] Hays RD, Sherbourne CD, Mazel RM (1993). The RAND 36-Item Health Survey 1.0. Health Econ.

[41] Ware J, Kosinski M, Keller S (1994). SF-36 Physical and Mental Health Summary Scales: A User's Manual.

[42] Aaronson NK, Muller M, Cohen PD, Essink-Bot ML, Fekkes M, Sanderman R, Sprangers MA, te Velde A, Verrips E (1998). Translation, validation, and norming of the Dutch language version of the SF-36 Health Survey in community and chronic disease populations. J Clin Epidemiol.

[43] Rensen A, van Mol MM, Menheere I, Nijkamp MD, Verhoogt E, Maris B, Manders W, Vloet L, Verharen L (2017). Quality of care in the intensive care unit from the perspective of patient's relatives: development and psychometric evaluation of the consumer quality index 'R-ICU'. BMC Health Serv Res.

[44] van Mol MMC, Bakker EC, Nijkamp MD, Kompanje EJO, Bakker J, Verharen L (2014). Relatives' perspectives on the quality of care in an Intensive Care Unit: the theoretical concept of a new tool. Patient Educ Couns.

[45] Marshall G, Hays R (1994). The patient satisfaction questionnaire short-form (PSQ-18). Rand.

[46] Wechsler TF, Kümpers F, Mühlberger A (2019). Inferiority or Even Superiority of Virtual Reality Exposure Therapy in Phobias?-A Systematic Review and Quantitative Meta-Analysis on Randomized Controlled Trials Specifically Comparing the Efficacy of Virtual Reality Exposure to Gold Standard Exposure in Agoraphobia, Specific Phobia, and Social Phobia. Front Psychol.

[47] Kothgassner OD, Goreis A, Kafka JX, Van Eickels RL, Plener PL, Felnhofer A (2019). Virtual reality exposure therapy for posttraumatic stress disorder (PTSD): a meta-analysis. Eur J Psychotraumatol.

[48] Rogers JP, Chesney E, Oliver D, Pollak TA, McGuire P, Fusar-Poli P, Zandi MS, Lewis G, David AS (2020). Psychiatric and neuropsychiatric presentations associated with severe coronavirus infections: a systematic review and meta-analysis with comparison to the COVID-19 pandemic. Lancet Psychiatry.

[49] Nikayin S, Rabiee A, Hashem MD, Huang M, Bienvenu OJ, Turnbull AE, Needham DM (2016). Anxiety symptoms in survivors of critical illness: a systematic review and meta-analysis. Gen Hosp Psychiatry.

[50] Rabiee A, Nikayin S, Hashem MD, Huang M, Dinglas VD, Bienvenu OJ, Turnbull AE, Needham DM (2016). Depressive Symptoms After Critical Illness: A Systematic Review and Meta-Analysis. Crit Care Med.

[51] Pinkas J, Horowitz A (2020). Reducing Severity of Posttraumatic Stress Disorder in Intensive Care Unit Survivors. Dimens Crit Care Nurs.

[52] Fernandez-Gonzalo S, Turon M, De Haro C, López-Aguilar J, Jodar M, Blanch L (2018). Do sedation and analgesia contribute to long-term cognitive dysfunction in critical care survivors?. Med Intensiva (Engl Ed).

[53] Van Rompaey B, Elseviers MM, Schuurmans MJ, Shortridge-Baggett LM, Truijen S, Bossaert L (2009). Risk factors for delirium in intensive care patients: a prospective cohort study. Crit Care.

[54] Kavalnienė R, Deksnyte A, Kasiulevičius V, Šapoka V, Aranauskas R, Aranauskas L (2018). Patient satisfaction with primary healthcare services: are there any links with patients' symptoms of anxiety and depression?. BMC Fam Pract.

[55] Mukhopadhyay A, Song G, Sim PZ, Ting KC, Yoo JKS, Wang QL, Mascuri RBHM, Ong VHL, Phua J, Kowitlawakul Y (2016). Satisfaction Domains Differ between the Patient and Their Family in Adult Intensive Care Units. Biomed Res Int.

[56] Holanda Peña MS, Talledo NM, Ots Ruiz E, Lanza Gómez JM, Ruiz Ruiz A, García Miguelez A, Gómez Marcos V, Domínguez Artiga MJ, Hernández Hernández MÁ, Wallmann R, Llorca Díaz J, Proyecto HU-CI (2017). Satisfaction in the Intensive Care Unit (ICU). Patient opinion as a cornerstone. Med Intensiva.

[57] King J, O'Neill B, Ramsay P, Linden MA, Darweish Medniuk A, Outtrim J, Blackwood B (2019). Identifying patients' support needs following critical illness: a scoping review of the qualitative literature. Crit Care.

[58] Bethune G (1998). From Worst to First: Behind the Scenes of Continental's Remarkable Comeback.

