# Supplementary material for: Intensive Care Unit–Specific Virtual Reality for Critically Ill Patients With COVID-19: Multicenter Randomized Controlled Trial
Source: J Med Internet Res. 2022 Jan 31;24(1):e32368. doi: 10.2196/32368 (PMC8812141; doi:10.2196/32368)
Supplement: Multimedia Appendix 1 [file jmir_v24i1e32368_app1.pdf]

Multimedia Appendix 1

Translation of the novel questionnaire assessing ICU care and aftercare satisfaction and global rating, and the perspectives on the ICU-VR intervention

Supplement to:

Intensive Care Unit-specific Virtual Reality for Critically Ill COVID-19 Patients With COVID-19: Multicenter Randomized Controlled Trial.

Perspectives on ICU care and ICU aftercare and perspectives on the Intensive Care Unit-specific Virtual Reality intervention.

1. Perspectives on the added value of ICU-VR to ICU care and ICU aftercare

1) On a scale from 1 to 10, how satisfied were you with the provided **ICU care**?

1	2	3	4	5	6	7	8	9	10
☐	☐	☐	☐	☐	☐	☐	☐	☐	☐
Very unsatisfied				Neutral					Very satisfied

2) On a scale from 1 to 10, how would you value the provided **ICU care**?

1	2	3	4	5	6	7	8	9	10
☐	☐	☐	☐	☐	☐	☐	☐	☐	☐
Very unsatisfied				Neutral					Very satisfied

3) On a scale from 1 to 10, to what extent did the VR intervention improve your satisfaction with the provided **ICU care** (ONLY FOR PATIENTS IN THE ICU-VR GROUP)?

1	2	3	4	5	6	7	8	9	10
☐	☐	☐	☐	☐	☐	☐	☐	☐	☐
Not at all				Neutral					Very much

4) On a scale from 1 to 10, to what extent did the VR intervention, in your opinion, improve the quality of **ICU care** (ONLY FOR PATIENTS IN THE ICU-VR GROUP)?

1	2	3	4	5	6	7	8	9	10
☐	☐	☐	☐	☐	☐	☐	☐	☐	☐
Not at all				Neutral					Very much

5) On a scale from 1 to 10, how satisfied were you with the provided **ICU aftercare**?

1	2	3	4	5	6	7	8	9	10
☐	☐	☐	☐	☐	☐	☐	☐	☐	☐
Very unsatisfied				Neutral					Very satisfied

6) On a scale from 1 to 10, how would you value the provided **ICU aftercare**?

1	2	3	4	5	6	7	8	9	10
☐	☐	☐	☐	☐	☐	☐	☐	☐	☐
Very unsatisfied				Neutral					Very satisfied

7) On a scale from 1 to 10, to what extent did the VR intervention improve your satisfaction with the provided **ICU aftercare** (ONLY FOR PATIENTS IN THE ICU-VR GROUP)?

1	2	3	4	5	6	7	8	9	10
☐	☐	☐	☐	☐	☐	☐	☐	☐	☐
Very unsatisfied				Neutral					Very satisfied

8) On a scale from 1 to 10, to what extent did the VR intervention, in your opinion, improve the quality of the **provided ICU aftercare** (ONLY FOR PATIENTS IN THE ICU-VR GROUP)?

1	2	3	4	5	6	7	8	9	10
☐	☐	☐	☐	☐	☐	☐	☐	☐	☐
Not at all				Neutral					Very much

2. Perspectives on the timing and number of sessions
(ONLY FOR PATIENTS IN THE ICU-VR GROUP)

9) Would you have like to see the intervention multiple times?

Yes	No	I don't know
☐	☐	☐

10) Would you have liked to be given the possibility to see the intervention at home as well?

Yes	No	I don't know
☐	☐	☐

11) Do you think the timing of ICU-VR was appropriate?

Yes	No, earlier would have been better	No, later would have been better	I don't know
☐	☐	☐	☐

3. Overall perspective on the ICU-VR intervention
(ONLY FOR PATIENTS IN THE ICU-VR GROUP)

12) On a scale from 1 to 10, how much would you recommend other ICU survivors to use the VR intervention?

1	2	3	4	5	6	7	8	9	10
☐	☐	☐	☐	☐	☐	☐	☐	☐	☐
Not at all				Neutral					Very much

13) On a scale from 1 to 10, how happy are you that you have seen the VR intervention?

1	2	3	4	5	6	7	8	9	10
☐	☐	☐	☐	☐	☐	☐	☐	☐	☐
Not at all				Neutral					Very much

14) On a scale from 1 to 10, how would you value the ICU-VR intervention in general?

1	2	3	4	5	6	7	8	9	10
☐	☐	☐	☐	☐	☐	☐	☐	☐	☐
Very bad				Neutral					Very good

4. Perspectives on the content of the ICU-VR intervention.
(ONLY FOR PATIENTS IN THE ICU-VR GROUP)

15) On a scale from 1 to 10, to what extent did the VR intervention fulfill your needs for information about your ICU treatment?

1	2	3	4	5	6	7	8	9	10
☐	☐	☐	☐	☐	☐	☐	☐	☐	☐
Not at all				Neutral					Very much

16) On a scale from 1 to 10, how comprehensive did you find the explanation given in the intervention?

1	2	3	4	5	6	7	8	9	10
☐	☐	☐	☐	☐	☐	☐	☐	☐	☐
Not at all				Neutral					Very much

17) On a scale from 1 to 10, to what extent did you think the information given was complete?

1	2	3	4	5	6	7	8	9	10
☐	☐	☐	☐	☐	☐	☐	☐	☐	☐
Not at all				Neutral					Very much

5. Perspectives on the effect of the ICU-VR intervention.
(ONLY FOR PATIENTS IN THE ICU-VR GROUP)

18) On a scale from 1 to 10, how much did the VR intervention help you understand what happened to you during your ICU treatment?

1	2	3	4	5	6	7	8	9	10
☐	☐	☐	☐	☐	☐	☐	☐	☐	☐
Not at all				Neutral					Very much

19) On a scale from 1 to 10, to what extent did the VR intervention help you understand your memories from the ICU treatment, or did it help you put those memories into perspective?

1	2	3	4	5	6	7	8	9	10
☐	☐	☐	☐	☐	☐	☐	☐	☐	☐
Not at all				Neutral					Very much

20) On a scale from 1 to 10, to what extent did the VR intervention help you put your ICU admission at rest?

1	2	3	4	5	6	7	8	9	10
☐	☐	☐	☐	☐	☐	☐	☐	☐	☐
Not at all				Neutral					Very much

21) On a scale from 1 to 10, to what extent did the VR intervention help you process your ICU admission emotionally?

1	2	3	4	5	6	7	8	9	10
☐	☐	☐	☐	☐	☐	☐	☐	☐	☐
Not at all				Neutral					Very much
