# Supplementary material for: Intensive Care Unit–Specific Virtual Reality for Critically Ill Patients With COVID-19: Multicenter Randomized Controlled Trial
Source: J Med Internet Res. 2022 Jan 31;24(1):e32368. doi: 10.2196/32368 (PMC8812141; doi:10.2196/32368)
Supplement: Multimedia Appendix 2 [file jmir_v24i1e32368_app2.pdf]

Multimedia Appendix 2

Table S1. Outcomes of the individual EQ-5D domains.

Supplement to:

Intensive Care Unit-specific Virtual Reality for Critically Ill COVID-19 Patients With COVID-19: Multicenter Randomized Controlled Trial.

Table S1. Outcomes of the individual EQ-5D domains.				
**Baseline** (3 months after hospital discharge)		ICU-VR group	Control group	*P*-value
	No problems	19 (43%)	17 (40%)	
	Slight problems	11 (25%)	13 (30%)	
Mobility	Moderate problems	13 (30%)	10 (23%)	
	Severe problems	1 (2%)	2 (5%)	
	Extreme problems	0 (0%)	1 (2%)	
	No problems	30 (68%)	25 (58%)	
	Slight problems	10 (27%)	11 (26%)	
Self-care	Moderate problems	4 (9%)	6 (26%)	
	Severe problems	0 (0%)	0 (0%)	
	Extreme problems	0 (0%)	1 (2%)	
	No problems	21 (47%)	12 (28%)	
	Slight problems	8 (18%)	10 (23%)	
Daily activities	Moderate problems	9 (20%)	12 (28%)	
	Severe problems	4 (9%)	8 (19%)	
	Extreme problems	2 (5%)	1 (2%)	
	No problems	10 (23%)	10 (23%)	
	Slight problems	14 (32%)	11 (26%)	
Pain / Discomfort	Moderate problems	18 (41%)	12 (28%)	
	Severe problems	2 (5%)	9 (21%)	
	Extreme problems	0 (0%)	1 (2%)	
	No problems	28 (64%)	17 (40%)	
	Slight problems	11 (25%)	15 (35%)	
Anxiety / Depression	Moderate problems	4 (9%)	7 (16%)	
	Severe problems	1 (2%)	3 (7%)	
	Extreme problems	0 (0%)	1 (2%)	
4 months after hospital discharge				
	No problems	18 (41%)	17 (40%)	
	Slight problems	12 (27%)	10 (23%)	
Mobility	Moderate problems	12 (27%)	13 (30%)	.712
	Severe problems	2 (5%)	3 (7%)	
	Extreme problems	0 (0%)	0 (0%)	
	No problems	34 (77%)	25 (58%)	
	Slight problems	8 (18%)	11 (26%)	
Self-care	Moderate problems	2 (5%)	7 (16%)	.119
	Severe problems	0 (0%)	0 (0%)	
	Extreme problems	0 (0%)	0 (0%)	
	No problems	14 (32%)	11 (26%)	
	Slight problems	19 (42%)	16 (37%)	
Daily activities	Moderate problems	8 (18%)	10 (23%)	.512
	Severe problems	3 (7%)	5 (12%)	
	Extreme problems	0 (0%)	1 (2%)	

Pain / Discomfort	No problems	8 (18%)	9 (21%)	
	Slight problems	20 (45%)	18 (42%)	
	Moderate problems	13 (30%)	12 (28%)	.596
	Severe problems	3 (7%)	4 (9%)	
	Extreme problems	0 (0%)	0 (0%)	
Anxiety / Depression	No problems	25 (57%)	20 (47%)	
	Slight problems	10 (23%)	15 (35%)	
	Moderate problems	6 (14%)	6 (14%)	.389
	Severe problems	1 (2%)	2 (5%)	
	Extreme problems	2 (5%)	0 (0%)	
6 months after hospital discharge				
Mobility	No problems	22 (56%)	16 (43%)	
	Slight problems	11 (28%)	9 (24%)	
	Moderate problems	6 (15%)	10 (27%)	.227
	Severe problems	0 (0%)	2 (5%)	
	Extreme problems	0 (0%)	0 (0%)	
Self-care	No problems	34 (87%)	25 (68%)	
	Slight problems	2 (5%)	9 (24%)	
	Moderate problems	3 (8%)	3 (8%)	.114
	Severe problems	0 (0%)	0 (0%)	
	Extreme problems	0 (0%)	0 (0%)	
Daily activities	No problems	19 (49%)	8 (22%)	
	Slight problems	14 (36%)	15 (41%)	
	Moderate problems	4 (10%)	9 (24%)	.149
	Severe problems	2 (5%)	5 (14%)	
	Extreme problems	0 (0%)	0 (0%)	
Pain / Discomfort	No problems	13 (33%)	10 (27%)	
	Slight problems	20 (51%)	13 (35%)	
	Moderate problems	4 (10%)	11 (30%)	.886
	Severe problems	2 (5%)	3 (8%)	
	Extreme problems	0 (0%)	0 (0%)	
Anxiety / Depression	No problems	28 (72%)	23 (62%)	
	Slight problems	8 (21%)	6 (16%)	
	Moderate problems	2 (5%)	6 (16%)	.198
	Severe problems	1 (3%)	2 (5%)	
	Extreme problems	0 (0%)	0 (0%)	

Outcomes of the five domains of the EQ-5D. Values are presented as absolute number (relative frequency) of patients reporting no, slight, moderate, severe, or extreme problems. P-values were calculated using a logistic mixed model, with randomization and baseline score as independent variables.
