# Supplementary material for: Intensive Care Unit–Specific Virtual Reality for Critically Ill Patients With COVID-19: Multicenter Randomized Controlled Trial
Source: J Med Internet Res. 2022 Jan 31;24(1):e32368. doi: 10.2196/32368 (PMC8812141; doi:10.2196/32368)
Supplement: Multimedia Appendix 3 [file jmir_v24i1e32368_app3.pdf]

Multimedia Appendix 3

Table S2. Subscales of the SF-36 throughout follow-up.

Supplement to:

Intensive Care Unit-specific Virtual Reality for Critically Ill COVID-19 Patients With COVID-19: Multicenter Randomized Controlled Trial.

Table S2. Subscales of the SF-36 throughout follow-up.

Baseline (3 months after hospital discharge)		ICU-VR group	Control group	*P*-value
Physical functioning		70 (54-81)	55 (35-80)	
Social functioning		75 (50-100)	63 (38-88)	
Physical role functioning		25 (0-100)	0 (0-50)	
Emotional role functioning		100 (33-100)	67 (0-100)	
Mental health		84 (68-92)	68 (52-84)	
Vitality		60 (45-76)	45 (35-65)	
Bodily pain		69 (53-83)	67 (45-78)	
General health perception		55 (40-70)	45 (35-65)	
Health change	Much worse (0)	14 (32%)	16 (39%)	
Compared to one year	Somewhat worse (25)	19 (43%)	13 (32%)	
ago, how would you	About the same (50)	5 (11%)	7 (17%)	
rate your general	Somewhat better (75)	5 (11%)	3 (7%)	
health now?	Much better (100)	1 (2%)	2 (5%)	
**4 months after hospital discharge**				
Physical functioning		70 (55-80)	55 (30-78)	.011
Social functioning		69 (50-88)	63 (38-88)	.738
Physical role functioning		25 (0-56)	25 (0-63)	.370
Emotional role functioning		83 (33-100)	67 (0-100)	.485
Mental health		76 (64-88)	68 (52-88)	.174
Vitality		55 (49-70)	50 (38-73)	.565
Bodily pain		68 (58-78)	68 (45-84)	.673
General health perception		55 (40-65)	45 (35-65)	.341
	Much worse (0)	12 (27%)	14 (33%)	
	Somewhat worse (25)	17(39%)	18 (42%)	
Health change	About the same (50)	9 (20%)	9 (21%)	.820
	Somewhat better (75)	4 (9%)	1 (2%)	
	Much better (100)	2 (5%)	1 (2%)	
**6 months after hospital discharge**				
Physical functioning		80 (58-95)	65 (45-85)	.222
Social functioning		100 (63-100)	75 (47-88)	.146
Physical role functioning		25 (0-100)	0 (0-75)	.607
Emotional role functioning		100 (67-100)	67 (0-100)	.118
Mental health		84 (70-92)	72 (56-88)	.185
Vitality		70 (45-78)	60 (35-70)	.878
Bodily pain		78 (67-90)	68 (55-90)	.445
General health perception		60 (43-73)	50 (35-65)	.478
	Much worse (0)	7 (18%)	13 (35%)	
	Somewhat worse (25)	14 (36%)	15 (41%)	
Health change	About the same (50)	11 (28%)	6 (16%)	.166
	Somewhat better (75)	4 (10%)	1 (3%)	
	Much better (100)	3 (8%)	2 (5%)	

All values are presented as median (IQR) score, except health change, which is presented as absolute number (relative frequency) of patients reporting their health to be much worse, somewhat worse, about the same, somewhat better, or much better than one year ago. *P*-values were calculated using linear (physical role functioning, social functioning, physical role functioning, social role functioning, emotional well-being, vitality, bodily pain, and general health perception) or logistic (health change) mixed models, with randomization and baseline value as independent variables.
