# Supplementary material for: Intensive Care Unit–Specific Virtual Reality for Critically Ill Patients With COVID-19: Multicenter Randomized Controlled Trial
Source: J Med Internet Res. 2022 Jan 31;24(1):e32368. doi: 10.2196/32368 (PMC8812141; doi:10.2196/32368)
Supplement: Multimedia Appendix 4 [file jmir_v24i1e32368_app4.pdf]

Multimedia Appendix 4

Table S3. Perspectives on the ICU-VR intervention.

Supplement to:

Intensive Care Unit-specific Virtual Reality for Critically Ill COVID-19 Patients With COVID-19: Multicenter Randomized Controlled Trial.

Table S3. Perspectives on the ICU-VR intervention.

Perspectives on the timing and number of sessions, n (%)			
			ICU-VR group
1)	Would you have liked to see the intervention multiple times?	Yes	11 (30%)
		No	19 (51%)
		I don't know	7 (19%)
2)	Would you have liked to be given the possibility to see the intervention at home as well?	Yes	14 (38%)
		No	22 (59%)
		I don't know	1 (3%)
3)	Do you think the timing was appropriate?	Yes	27 (73%)
		No, earlier would have been better	7 (19%)
		No, later would have been better	0 (0%)
		I don't know	3 (8%)
**Overall perspectives on the ICU-VR intervention, mean (SD)**			
4)	On a scale from 1 to 10, how much would you recommend other ICU survivors to use the VR intervention?	Score	9.0 (1.1)
		Score >5, n (%)	37 (100%)
5)	On a scale from 1 to 10, how happy are you that you have seen the VR intervention?	Score	8.8 (1.4)
		Score >5, n (%)	35 (95%)
6)	On a scale from 1 to 10, how would you value the ICU-VR intervention in general?	Score	8.7 (1.0)
		Score >5, n (%)	37 (100%)
**Perspectives on the content of the ICU-VR intervention, mean (SD)**			
7)	On a scale from 1 to 10, to what extent did the VR intervention fulfill your needs for information?	Score	6.8 (2.5)
		Score >5, n (%)	28 (76%)
8)	On a scale from 1 to 10, how comprehensive did you find the explanation given in the intervention?	Score	8.6 (1.2)
		Score >5, n (%)	36 (97%)
9)	On a scale from 1 to 10, to what extent did you think the information given was complete?	Score	8.4 (1.4)
		Score >5, n (%)	35 (95%)
**Perspectives on the effect of the ICU-VR intervention, mean (SD)**			
10)	On a scale from 1 to 10, how much did the VR intervention help you understand what happened to you during your ICU treatment?	Score	7.2 (2.5)
		Score >5, n (%)	28 (76%)
11)	On a scale from 1 to 10, to what extent did the VR intervention help you understand your memories from the ICU treatment, of did it help you put those memories into perspective?	Score	6.6 (2.8)
		Score >5, n (%)	24 (65%)
12)	On a scale from 1 to 10, to what extent did the VR intervention help you put your ICU admission at rest?	Score	6.8 (2.8)
		Score >5, n (%)	27 (73%)
13)	On a scale from 1 to 10, to what extent did the VR intervention help you process your ICU admission emotionally?	Score	6.2 (3.0)
		Score >5, n (%)	24 (65%)

Outcomes of the perspectives on ICU-VR questionnaire. These questions were only filled out by patients randomized to the ICU-VR group.
